# Neutrophil count is associated with myeloid derived suppressor cell level and presents prognostic value for hepatocellular carcinoma patients

**DOI:** 10.18632/oncotarget.15456

**Published:** 2017-02-17

**Authors:** Xing Li, Yan-Fang Xing, Ai-Hua Lei, Qiang Xiao, Zhi-Huan Lin, Ying-Fen Hong, Xiang-Yuan Wu, Jie Zhou

**Affiliations:** ^1^ Program in Immunology, Affiliated Guangzhou Women and Children's Medical Center, Zhongshan School of Medicine, Sun Yat-Sen University, Guangzhou, China; ^2^ Institute of Human Virology, Zhongshan School of Medicine, Sun Yat-Sen University, Guangzhou, China; ^3^ Department of Medical Oncology and Guangdong Key Laboratory of Liver Disease, the Third Affiliated Hospital of Sun Yat-Sen University, Guangzhou, China; ^4^ Department of Nephrology, the Third Affiliated Hospital of Guangzhou Medical University, Guangzhou, China

**Keywords:** neutrophil counts, hepatocellular carcinoma, patient selection, prognosis, myeloid derived suppressor cell

## Abstract

Myeloid Derived Suppressor Cell (MDSC) has been raised to be a novel target for multiple cancers. However, target agents on MDSC have not display promising efficacy. One of the critical reasons shall be less optimal patient selection. In the present study, we aimed to identify clinical parameters relevant to MDSC level in hepatocellular carcinoma (HCC) patients for future MDSC targeted therapy. In the present study, a series of 55 HCC patients (testing group) and 20 healthy donors were analyzed investigating frequencies of MDSC in peripheral blood mononuclear cells (PBMC). As a result, we found that MDSC level was increased in HCC patients compared to healthy donors (10.33% vs 1.54%, *p* < 0.0001). The monocytes (r^2^ = 0.2875, *p* < 0.0001), neutrophils (r^2^ = 0.3630, *p* < 0.0001) and platelet counts (r^2^ = 0.0828, *p* = 0.0331) in circulation was positively associated with MDSC level. Then, the prognostic value of the above predictors was determined in a retrospective database of 255 HCC patients (validation group). The baseline characteristics of testing and validation group were similar. Multivariate analysis by Cox regression revealed that neutrophil count was an independent predictor for overall survival (OS) (*p* = 0.000, HR 1.065, 95% CI 1.028–1.103), with the rest parameters failed to reach a significant result. In summary, the present study firstly identified blood neutrophil counts was a predictor of MDSC level in PBMC for HCC patients. And, patients with higher neutrophil count level might be the optimal patient subgroup for MDSC targeted therapy.

## INTRODUCTION

Myeloid Derived Suppressor Cell (MDSC) has been raised to be a novel target and a prognostic factor for many malignant diseases, especially for hepatocellular carcinoma (HCC) [1–6]. However, target agents on MDSC have not display promising efficacy for HCC patients. One of the critical reasons shall be the heterogeneity of patients which made patient selection very complicated for the success of clinical trials. However, there is no investigation on the patient selection criteria for MDSC based clinical trials. Directly testing MDSC level in HCC patients might be a plausible way to select optimal patients. However, it is not easy to standardize procedure and MDSC testing among different researchers and institutions [7], which make multicenter clinical trials less practical. Thus, it is necessary to identify clinically practical parameters as specific patient selection criteria.

Neutrophil, monocyte and platelet count in blood was reported to be promising prognostic factors for HCC patients by a series of studies including ours [8–11], which made them potential patient selection criteria for clinical trials. However, the mechanism was not identified yet. Interestingly, neutrophils in blood consisted of neutrophils and MDSC [12, 13]. Thus, neutrophils count might reflect the MDSC level of HCC patients and be a practical selection criterion for multicenter clinical trials.

In the present study, we investigated the circulation MDSC levels and their association with clinical parameters of peripheral blood cell counts. Finally, the prognostic value of MDSC related clinical parameters were tested by integrated into CLIP score system. The present study aimed to identify a promising patient selection criterion for MDSC targeted treatments.

## RESULTS

### PMN-MDSCs and M-MDSCs level were elevated in HCC patients

**Figure 1:**
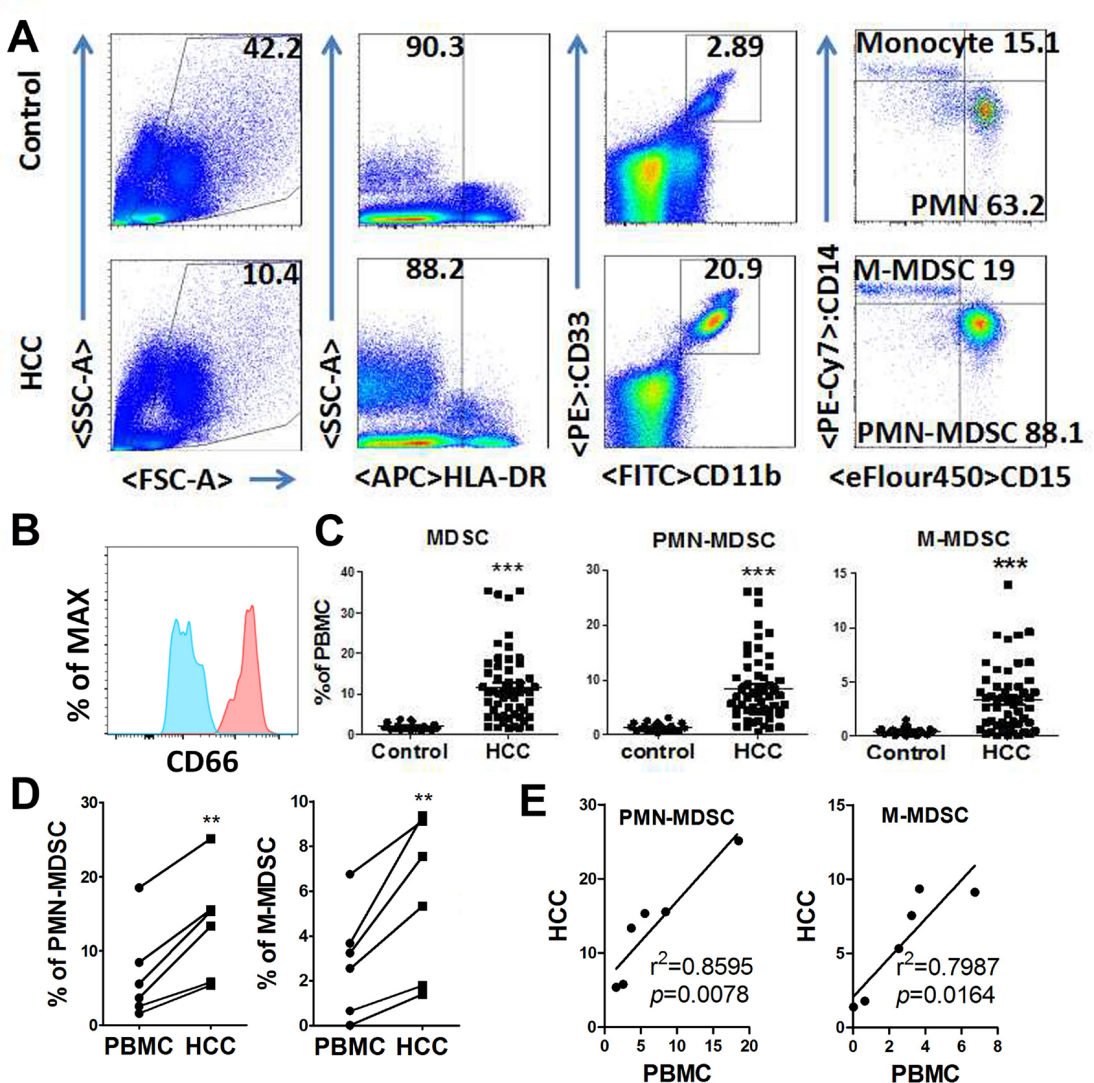
Expansion of PMN-MDSC and M-MDSC in HCC patients (**A**) Gating strategy of PMN-MDSC/M-MDSC by flow cytometry analysis. PMN-MDSC was defined as HLA-DR^−/low^CD11b^+^CD33^+^CD14^−^CD15^+^, with M-MDSC defined as HLA-DR^−/low^ CD11b^+^CD33^+^CD14^+^CD15^−^. (**B**) CD66 expression in PMN-MDSCs (red) and M-MDSC (blue). (**C**) Statistical analysis of PMN-MDSC and M-MDSC frequency in the peripheral blood from HCC patients and healthy controls. (**D**) Comparison of PMN-MDSC/M-MDSC level in PBMC and tumor tissue. (**E**) Association of PMN-MDSC/M-MDSC level in PBMC and tumor tissue by Linear regression. **P < 0.01; ***P < 0.001.

A total of 55 HCC patients and 20 aged and sex match controls were tested PMN-MDSC (HLA-DR^−/low^ CD11b^+^CD33^+^CD14^−^CD15^+^), M-MDSC (HLA-DR^−/low^ CD11b^+^CD33^+^CD14^+^CD15^−^) level in peripheral blood mononuclear cells (PBMCs) [13] Figure 1A. PMN-MDSCs were CD66b^+^ and M-MDSCs were CD66b^−^. (Figure 1B). As a result, we found that total MDSC level in HCC patients (median 10.33%, range 1.43–35.34%) were significantly increased compared with health control (median 1.54%, range 0.91–3.76%). PMN-MDSC level (median 6.74%, range 0.60–26.03%) was increased in HCC patients compared to healthy donors (median 1.35%, range 0.40–3.13%). M-MDSC level was also higher in HCC group (median 2.79 %, range 0.00–13.99%) than the healthy control (median 0.44%, range 0.00–1.48%). (Figure 1C).

MDSCs in tumor were relatively higher than PBMC. And, the results were parallel between PMN-MDSC and M-MDSC (Figure 1D). Notably, MDSC and PMN-MDSC levels were positively related to those in PBMC (Figure 1E), with M-MDSC levels reached a marginal positive result.

**Figure 2:**
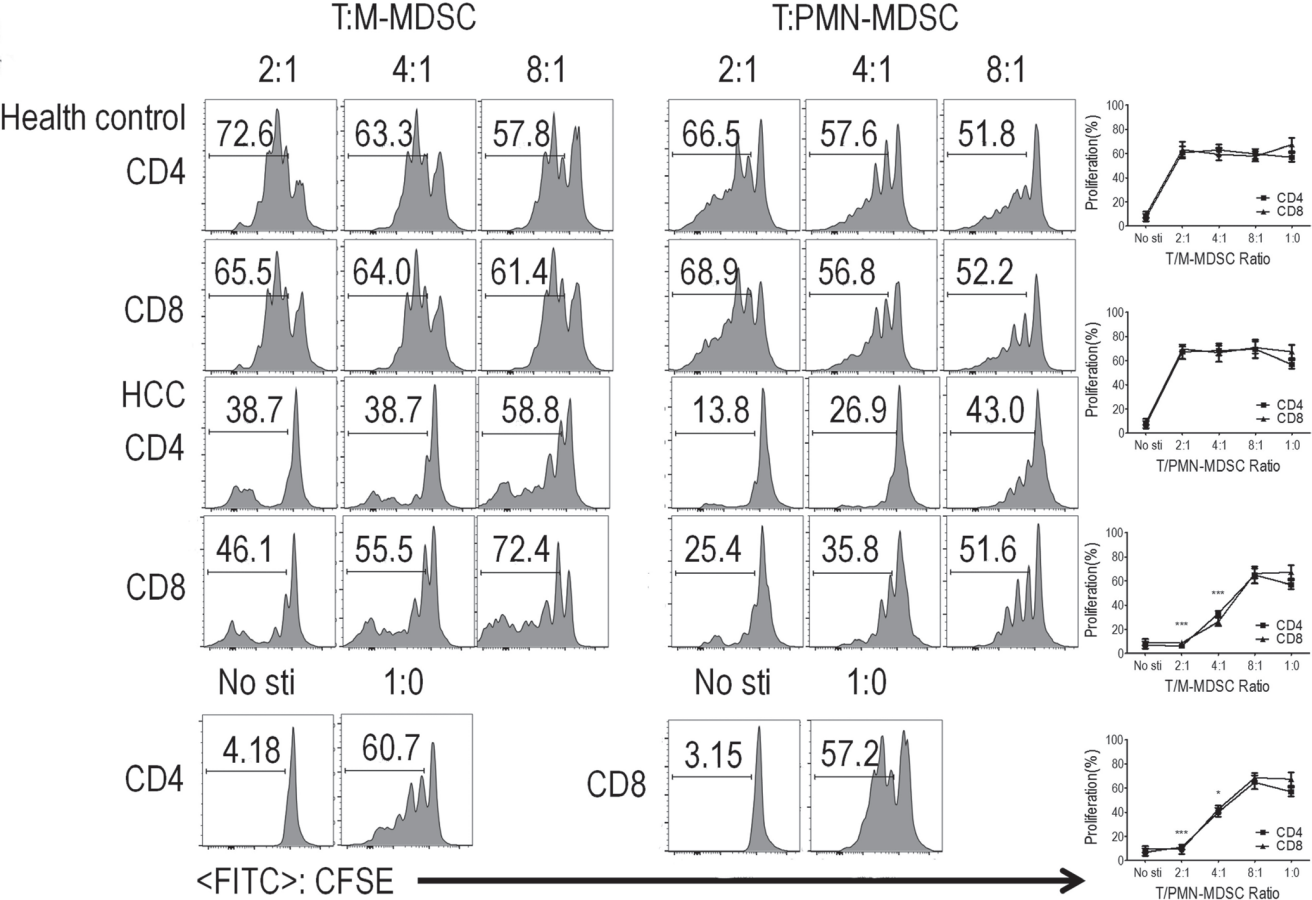
PMN-MDSC and M-MDSC from HCC patients and health donor suppressed T cell proliferation and activation CD3+ T cells from PBMCs were stimulated with anti-CD3 and anti-CD28, co-cultured with PMN-MDSCs/M-MDSC from the same donors at different ratios for 3 days, and evaluated for CD4+ and CD8+ T cell proliferation by CFSE labeling. Left panels: Representative flow cytometry data from 1 individual. Right panels: Cumulative data. Bottom: Representative flow cytometry data of positive control and negative control. (*n* = 5). *P < 0.05; ***P < 0.001.

In order to confirm immune suppressive capacity of PMN-MDSC/M-MDSC in HCC patients, T cells and PMN-MDSC or M-MDSC were purified from PBMC using Flow sorting, respectively. CFSE-labeled PBMC-derived CD3+ T cells were stimulated with anti-CD3/anti-CD28 with or without PMN-MDSC or M-MDSC. CD4+ and CD8+ T cell proliferation were abrogated by the addition of PMN-MDSC or M-MDSC with a dosage dependent manner. (Figure 2).

### PMN-MDSCs and M-MDSCs levels were associated with multiple parameters of peripheral blood cell counts

**Figure 3:**
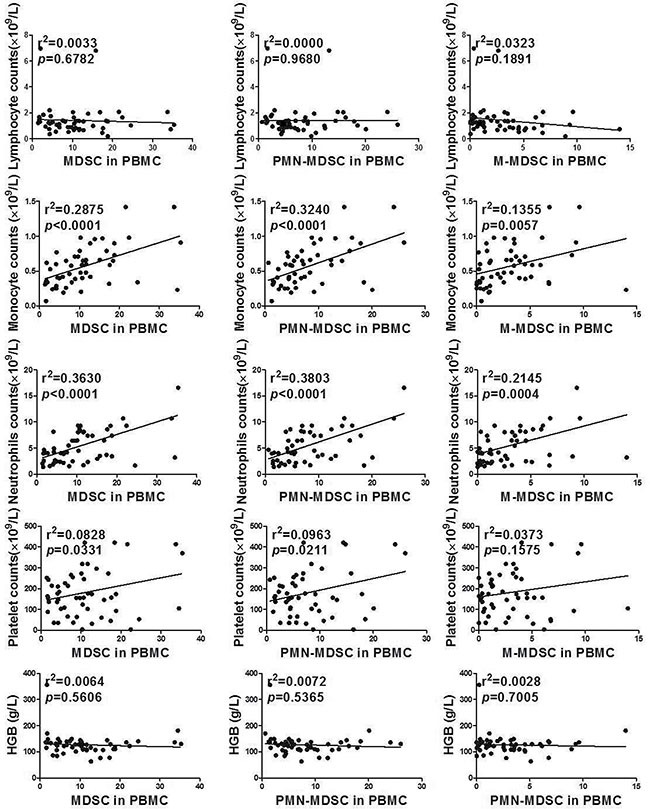
Association between MDSC/PMN-MDSC/M-MDSC and clinical parameters of peripheral blood cell counts by Linear regression

The association between clinical parameters of peripheral blood cell counts and MDSC level were tested by Linear regression. MDSC levels were associated with monocytes (r^2^ = 0.2875, *p* < 0.0001), neutrophils (r^2^ = 0.3630, *p* < 0.0001) and platelet counts (r^2^ = 0.0828, *p* = 0.0331) in circulation significantly. Specifically, PMN-MDSC levels positively related to monocytes (r^2^ = 0.3240, *p* < 0.0001), neutrophils (r^2^ = 0.3803, *p* < 0.0001) and platelet counts (r^2^ = 0.0963, *p* = 0.0211) in circulation significantly. Similarly, M-MDSC levels displayed positive association with monocytes (r^2^ = 0.1355, *p* = 0.0057) and neutrophils (r^2^ = 0.2145, *p* = 0.0004). (Figure 3)

### Prognostic value of clinical parameters related to MDSC levels

In order to determine the prognostic value of MDSC related clinical parameters, a retrospective consisted of 245 HCC patients were utilized. The baseline characteristics of the validation group were similar to the testing group (Table 1). The prognostic value for overall survival (OS) of the clinical parameters related to MDSC were tested by integrated into Cancer of the Liver Italian Program (CLIP) score system, a well acknowledged prognostic score system for HCC patients [8, 10, 14]. Multivariate analysis by Cox regression revealed that neutrophils were associated with unfavorable OS (*p* = 0.000, HR 1.065, 95% CI 1.028–1.103), with the rest parameters failed to reach a significant result (Table 2). Besides, neutrophil count significantly correlated with tumor length (*p* = 0.000), portal vein thrombosis (*p* = 0.036), lymph node metastasis (*p* = 0.032), TNM7th stage (*p* = 0.000) and AFP (*p* = 0.002). Since neutrophils count well acknowledged as a prognostic factors in HCC patients, its association with MDSC level shall be the latent mechanism, which indicated the prognostic value of MDSC.

**Table 1:** Baseline demographic and clinical characteristics of hepatocellular carcinoma patients in testing group and validation group

Characteristics	Testing Group (*n* = 55)	Validation Group (*n* = 245)	
Gender			0.175
Male	52 (94.5%)	213 (86.9%)	
Female	3 (5.5%)	32 (13.1%)	
Age (years)	56.4 (32.0∼79.0)	57.0 (19.0–86.0)	0.468
HBsAg	48 (87.3%)	214 (87.3%)	0.999
Liver cirrhosis	41 (74.5%)	183 (74.7%)	0.999
MDSC(%)	10.33 (1.43–35.34)		
PMN-MDSC(%)	6.74 (0.60–26.03)		
M-MDSC(%)	2.79 (0.00–13.99)		
Laboratory parameters			
International normalized ratio	1.16 (0.95∼4.01)	1.17 (0.85–2.53)	0.901
AFP (ng/mL)	274 (1.73∼121000)	708.6 (1–212620)	0.517
Albumin (g/L)	35.4 (24.0∼54.0)	34.6 (25.2–51.8)	0.193
ALP (U/L)	129 (50∼1591)	149 (33–767)	0.331
Fibrinogen (g/L)	2.73 (1.11∼5.67)	3.05 (0.90–9.40)	0.534
Total bilirubin (μmol/L)	24.3 (9.2∼540)	35.7 (5–865)	0.278
ALT (U/L)	51 (10∼1284)	55 (4–2669)	0.193
Platelets (×10^9^/L)	164 (4∼421)	141 (4–503)	0.180
Hemoglobin (g/L)	126 (63∼356)	119 (56–177)	0.086
Monocyte (×10^9^/L)	0.58 (0.07∼1.42)	0.60 (0.02–2.95)	0.121
Neutrophil (×10^9^/L)	4.5 (1.4∼16.6)	4.3 (0.5–30.6)	0.264
Lymphocyte (×10^9^/L)	1.22 ( 0.20∼6.96)	1.17 (0.32–14.0)	0.829
TNM 7^th^Edition			0.07
I	13 (23.6%)	30 (12.2%)	
II	8 (14.5%)	21 (8.6%)	
III	21 (56.4%)	140 (57.1%)	
IV	3 (5.5%)	54 (22.0%)	
CLIP score			0.067
0	7 (12.7%)	11 (4.5%)	
1	7 (12.7%)	23 (9.4%)	
2	4 (7.3%)	39 (15.9%)	
3	11 (20.0%)	41 (16.7%)	
4	14 (25.5%)	60 (24.5%)	
5	8 (14.5%)	62 (25.3%)	
6	4 (7.3%)	9 (3.7%)	

Abbreviations: AFP, α-fetoprotein; ALP, alkaline phosphatase; ALT, alanineaminotransferase; CLIP, Cancer of the Liver Italian Program.

**Table 2:** Multivariate Analysis and Integration into CLIP of the prognostic factors for OS associated with MDSC level among HCC patients

Characteristics	*P*	HR	95% CI for HR
CLIP	0.000	1.529	1.385–1.688
Neutrophil	0.000	1.065	1.028–1.103
Lymphocyte	0.895	0.988	0.825–1.184
Platelets	0.454	1.001	0.999–1.002
Hemoglobin	0.413	0.997	0.991–1.004
Monocyte	0.702	0.930	0.641–1.349

Abbreviation: CLIP, Cancer of the liver Italian Program scoring system for hepatocelluar carcinoma; OS, overall survival; HCC, hepatocellular carcinoma; HR, hazardsratio; 95% CI,95% confidence interval.

## DISCUSSION

In this era of immunotherapy, patient selection for clinical trials on novel targets has become a top issue for presenting efficacy [15, 16]. Myeloid derived suppressor cell (MDSC) has been raised to be a novel target for multiple cancers with dozens of clinical available agents developed [12, 17]. However, target agents on MDSC has not displayed promising efficacy in major cancer types including HCC. One of the critical reasons shall be less optimal patient selection. HCC is a highly heterogeneous disease [18–20], which makes patients selection more complicated. MDSC testing for patients was not easily to be standard between investigators and institutions [7], thus it was imperative to find clinically feasible parameters that were highly relevant to MDSC level. Then patient selection for MDSC targeted therapy might be facilitated by specific parameters. In the present study, we identified blood neutrophils count were relevant to MDSC level and a strong prognostic factor for HCC patients. Future clinical trials investigating MDSC targeted therapy might include neutrophil count as one of the criteria.

Our series studies and many reports revealed that neutrophils count was an independent prognostic factor for HCC patients[8]. However, the underlining mechanism was far from identified. Some studies suggested that the neutrophil levels were related to the systematic release of chemokines and ILs, including vascular endothelial growth factor [21], angiopoietin-1 [22] and matrix metalloproteinases-9 [23], which promoted tumor growth and metastasis in HCC. Besides, the presence of neutrophils in the tumor stroma was associated with a poor prognosis[24]. In the present study, we proposed a more reasonable explanation, which was neutrophils count positively associated with MDSC level, which was a well known promoter of malignant diseases.

PMN-MDSC were a subset of neutrophils with similar shape with normal ones [12, 23] and would be designated as neutrophils in routing blood test by automatic blood cell analyzer. Thus, neutrophils consisted of normal neutrophils and PMN-MDSCs. Elevation of PMN-MDSC had been confirmed in HCC patients by multiple studies [1–3]. The heterogeneity in PMN-MDSC levels among HCC patients decreased the accuracy of the patient selection for MDSCs targeted therapy. In the present study, we revealed that neutrophils count positively related to PMN-MDSC level, which indicated that utilization of neutrophil counts into patient selection might promote the accuracy of picking patients with optimal MDSC level.

Monocyte counts were relevant to M-MDSC levels in our study. M-MDSC presented similar morphology with monocytes [12, 23] and automatic blood cell analyzer shall identify it as monocytes. M-MDSC levels were slightly relevant to platelet count, which was a novel finding. Platelet count was considered a prognostic factor for HCC and an indicator of systematic inflammation [10]. The underling interaction between MDSC and platelet needs further investigation.

In summary, the present study firstly identified blood neutrophils counts was a predictor of MDSC level in PBMC for HCC patients. And, patients with higher neutrophils count level might be the optimal patient subgroup for MDSC targeted therapy.

## MATERIALS AND METHODS

### Patients and healthy donors

During the period between September 2014 and June 2015, we investigated a series of 55 advanced HCC patients who presented to the Third Affiliated Hospital of Sun Yat-sen University, Guangzhou, China (testing group). The diagnosis of HCC was confirmed by pathology or the American Association for the study of liver diseases radiological criteria by either computed tomography (CT) or magnetic resonance imaging (MRI). Age and gender matched healthy controls (*n* = 20) consisted of local volunteers. All patients and healthy control were also screened for serum human immunodeficiency virus (HIV) antibody, hepatitis B surface antigen (HBsAg), hepatitis C virus (HCV) antibody, hepatitis D virus (HDV) antigen and HDV antibody. Patients and healthy controls who were positive for HIV or chronic of hepatitis virus infection except for HBV, and who presented acute infections (including pneumonia, urinary tract infection and et al), who displayed pyrexial (temperature under the axillary is at or over37.2°C (99.0°F) within 1 week before admission, who were pregnant, patients who received systematic corticosteroids or immnuno-suppressive agents and those younger than 18 years old were excluded from thisstudy. None of the patients received any anti-cancer therapy before collection of PBMCs. This study was approved by the Clinical Ethics ReviewBoard of the Third Affiliated Hospital of Sun Yat-sen University. A written informed consent was obtained from all thepatients at the time of admission.

### Mononuclear cells in peripheral blood and tumor tissue isolation and flow cytometric analysis

Tissues were first digested with the Tumor Dissociation Kit, human AQ56 (Miltenyi), and then red blood cells were lysed. Mononuclear cells were isolated from whole blood or tissue suspension by Ficoll centrifugation and analyzed within 6 hour after blood sampling. The following anti-human antibodies were purchased from eBioscience(San Diego, CA): CD11b-fluorescein isothiocyanate (FITC), HLA-DR–APC, CD14-PE-Cy7, CD15-eFluor450, CD33-PE, CD33- PerCP-Cyanine5.5, CD66b-PE and their corresponding isotype controls. The cellphenotype was analyzed by flow cytometry on a flow cytometer FACSAria II flow cytometer (BD Bioscience), and data were analyzed with the FlowJo V10.0.7 (FlowJo, OR, USA).

### T cell proliferation assay

T cell proliferation was illustrated by 5,6-carboxyfluoresceindiacetate, succinimidylester (CFSE) dilution. Purified T cells were labeled with CFSE (3 μM; Invitrogen), stimulated with 0.5 μg/ml 3-h pre-coated anti-CD3 and 0.5 μg/ml anti-CD28 (eBioscience), and cultured alone or co-cultured with autologous PMN-MDSCs or M-MDSC at the indicated ratios for 3 days. The cells were then stained for surface marker expression with CD4-PE or CD8-PE-Cy5 antibodies, and T cell proliferation was analyzed on a flow cytometer. All cultures were conducted in the presence of 20 IU/ml recombinant human IL-2 (Miltenyi Biotec) in RPMI 1640 (Life Technologies) for 3–4 days at 37°C.

### Data collection

Institutional review board approval was obtained from the electronic charts needed to retrieve data regarding the potential prognostic factors, including age, sex, Karnofsky performance status (KPS), pre-therapy laboratory counts of neutrophils, lymphocytes, monocyte, hemoglobin, platelets and parameters included in Child-Pugh score and CLIP score and Seventh Edition AJCC tumor–node–metastasis (TNM) staging.

### Validation of the clinical parameters related to MDSC level as prognostic factors

In order to verify the prognostic value of the clinical parameters associated with MDSC level, a series of 245 consecutive HCC patients (validation group) were retrospectively collected and analyzed. The inclusion and exclusion criteria were the same the prospective group. The clinical parameters before anti-cancer treatment were collected and analyzed on their predictive value for OS. All patients underwent regular follow-up in our hospital. OS was calculated from the first day of treatment to the date of death from any cause.

### Statistics

The Kolmogorov-Smirnov test was used to evaluate the normality of distribution. Data were reported as median and range when distribution was not normal. Statistical differences in clinical characteristics between the 2 groups were compared using the *t*-test, the Mann-Whitney test or chi-square test. Group comparison tests were performed using the Wilcoxon rank-sum test. Linear regression was used to find correlation between clinical parameters and the MDSC levels. Multivariate analysis using a Logistic proportional hazards model was used to test for independent significance of all the clinical parameters relevant to MDSC level. The Spearman correlation was utilized to determine the association of clinical parameters. For all tests, a *p* value < 0.05 was considered statistically significant, and all *p* values quoted are 2-sided. Statistical analyses were performed using SPSS v. 20.0 (SPSS Inc., Chicago, IL, USA).
